# Investigation of brain iron in anorexia nervosa, a quantitative susceptibility mapping study

**DOI:** 10.1186/s40337-023-00870-4

**Published:** 2023-08-21

**Authors:** Parsa Ravanfar, R. Jarrett Rushmore, Amanda E. Lyall, Vanessa Cropley, Nikos Makris, Patricia Desmond, Dennis Velakoulis, Martha E. Shenton, Ashley I. Bush, Susan L. Rossell, Christos Pantelis, Warda T. Syeda, Andrea Phillipou

**Affiliations:** 1 Melbourne Neuropsychiatry Centre, Department of Psychiatry, The University of Melbourne and Royal Melbourne Hospital, Level 3, Alan Gilbert Building, 161 Barry ST, Carlton South, VIC 3053 Australia; 2 Psychiatry Neuroimaging Laboratory, Brigham and Women’s Hospital and Harvard Medical School, Boston, MA USA; 3 Center for Morphometric Analysis (CMA), Massachusetts General Hospital, Charlestown, MA USA; 4 Department of Anatomy and Neurobiology, Boston University School of Medicine, Boston, MA USA; 5 Department of Psychiatry, Brigham and Women’s Hospital, Harvard Medical School, Boston, MA USA; 6 Department of Psychiatry, Massachusetts General Hospital, Harvard Medical School, Boston, MA USA; 7 Department of Radiology, Royal Melbourne Hospital, University of Melbourne, Parkville, VIC Australia; 8 Neuropsychiatry, The Royal Melbourne Hospital, Parkville, VIC Australia; 9 Department of Radiology, Brigham and Women’s Hospital, and Harvard Medical School, Boston, MA USA; 10 Melbourne Dementia Research Centre, The Florey Institute of Neuroscience and Mental Health, The University of Melbourne, Parkville, VIC Australia; 11 Centre for Mental Health and Brain Sciences, Swinburne University, Hawthorn, VIC Australia; 12 Department of Mental Health, St Vincent’s Hospital, Melbourne, VIC Australia; 13 The Florey Institute of Neuroscience and Mental Health, The University of Melbourne, Parkville, VIC Australia; 14 Orygen, Melbourne, Australia; 15 Centre for Youth Mental Health, The University of Melbourne, Melbourne, Australia; 16 Department of Psychological Sciences, Swinburne University of Technology, Melbourne, Australia; 17 Department of Mental Health, Austin Health, Melbourne, Australia

**Keywords:** Anorexia nervosa, Magnetic resonance imaging, Brain, Iron, Quantitative susceptibility mapping

## Abstract

**Background:**

Anorexia nervosa (AN) is a potentially fatal psychiatric condition, associated with structural brain changes such as gray matter volume loss. The pathophysiological mechanisms for these changes are not yet fully understood. Iron is a crucial element in the development and function of the brain. Considering the systemic alterations in iron homeostasis in AN, we hypothesized that brain iron would be altered as a possible factor associated with structural brain changes in AN.

**Methods:**

In this study, we used quantitative susceptibility mapping (QSM) magnetic resonance imaging to investigate brain iron in current AN (c-AN) and weight-restored AN compared with healthy individuals. Whole-brain voxel wise comparison was used to probe areas with possible group differences. Further, the thalamus, caudate nucleus, putamen, nucleus accumbens, hippocampus, and amygdala were selected as the regions of interest (ROIs) for ROI-based comparison of mean QSM values.

**Results:**

Whole-brain voxel-wise and ROI-based comparison of QSM did not reveal any differences between groups. Exploratory analyses revealed a correlation between higher regional QSM (higher iron) and lower body mass index, higher illness severity, longer illness duration, and younger age at onset in the c-AN group.

**Conclusions:**

This study did not find evidence of altered brain iron in AN compared to healthy individuals. However, the correlations between clinical variables and QSM suggest a link between brain iron and weight status or biological processes in AN, which warrants further investigation.

**Supplementary Information:**

The online version contains supplementary material available at 10.1186/s40337-023-00870-4.

## Background

Anorexia nervosa (AN) is a mental illness characterized by distorted body image, overvalued ideation of having a large body habitus, and behaviors aimed at restricting food intake. AN is associated with the highest mortality rate among psychiatric disorders with a standardized mortality ratio of 5.9 [2]. Neuroimaging studies have reported adverse micro-/macrostructural and functional alterations in the brain, including gray matter volume loss, compromised white matter integrity, and widespread alterations in functional connectivity across the brain [15, 29, 30, 32, 33]. These changes are postulated to occur in association with both the primary neurobiological processes in AN [31] and the nutritional deficiencies that result from starvation [17]. Although the existing literature suggests a diverse array of structural brain alterations, the paucity of quantitative magnetic resonance imaging (MRI) studies to examine microstructural changes limits our understanding of the molecular and pathological processes that may contribute to these structural changes [17]. One of the potential microstructural changes that can be associated with the reported structural alterations in AN is the dysregulation of brain iron.

Iron is an essential cofactor for several vital neurophysiological processes that have been found to be impacted in AN. First, animal and human studies have shown that AN and starvation status are associated with mitochondrial fragmentation and dysfunction [41], increased oxidative stress [13, 21] and diminished cerebral glucose metabolism [1]. The mitochondrial oxidative phosphorylation complexes depend on iron as an electron buffer for aerobic energy metabolism [28]. Insufficient iron leads to mitochondrial dysfunction, and excess iron can facilitate the production of reactive oxygen species, generating to oxidative stress. Second, quantitative MRI studies have shown decreased myelin content in gray [6, 27] and white matter [37] in AN. Consistent with reduced myelin, diffusion-weighted imaging studies have reported lower fractional anisotropy in various white matter bundles suggesting diminished white matter integrity (for detailed review see [24]). Iron is an essential factor in myelin production by oligodendrocytes and its deficiency is associated with deficits in myelination [7, 22]. Third, iron is a key cofactor in dopamine and serotonin neurotransmission [5], which are aberrant in AN [11]. Iron deficiency impedes the physiologic function of these neurotransmitter systems and has been suggested to be associated with depressive and anxiety symptoms [35]. Considering the link between iron and the above-mentioned pathophysiologic changes observed in AN, it is reasonable to postulate regional brain iron content alterations in association with these changes.

There are only a few existing studies that have examined iron in AN. While serum iron levels are often normal in AN, marked increases in the concentrations of two important iron regulating proteins, ferritin and hepcidin, have been observed [8, 16, 26]. Hepcidin is an iron-regulating hormone secreted from the liver, which inhibits absorption of iron from the gut and its release from cells. Ferritin is the primary iron storage protein and reflects body’s iron storage. The exact mechanism for the increased levels of ferritin and hepcidin despite normal iron levels in AN is unclear, yet it suggests a perturbation in the systemic homeostasis of iron. Brain iron has not been investigated in human studies of AN. In our literature review, we identified one quantitative MRI study that used T2* relaxometry to examine myelin changes in AN [6]. However, in addition to myelin, T2* value is non-specifically sensitive to changes in iron and other metals. In that study, no difference in T2* signal was observed between AN and healthy control groups. The recent development of quantitative susceptibility mapping (QSM) allows for accurate voxel-level estimation of magnetic susceptibility as a proxy of tissue iron content, therefore, providing a reliable in vivo measurement of iron [19]. In this study, we used QSM for the first time to examine brain iron in individuals with current AN (c-AN) and weight-restored AN (wr-AN). The primary outcome measure was the comparison of magnetic susceptibility, as an indirect measure of brain iron, between groups. We hypothesized that both AN groups would demonstrate altered brain iron distribution compared to healthy individuals, which would be more pronounced in the c-AN group due to the concurrent effect of malnutrition. Based on the recent evidence suggesting a dynamic activity-dependent transport of iron within the brain [39] and the reported link between regional iron content and the severity and duration of illness reported in other neuropsychiatric conditions such as major depressive disorder and neurocognitive disorders [4, 34, 43], we explored the correlations between QSM and AN duration, age of onset, and severity of clinical symptoms as the secondary outcome measures.

## Methods

### Study design and patient population

In a cross-sectional study, from December 2016 to May 2019, fifty-four right-handed female participants older than 18 years (max. age = 37 years) were recruited through community advertisements. The c-AN group consisted of individuals with a current diagnosis of AN based on the Diagnostic and Statistical Manual of Mental Disorders, 5th edition (DSM-5) through the Mini International Neuropsychiatric Interview (MINI). Individuals with a past diagnosis of AN made by a mental health professional (psychiatrist or psychologist) who had maintained a body mass index (BMI) above 18.5 for a period of 12 months prior to assessments were included in the wr-AN group through outpatient visits. A group of healthy controls with no history of eating disorders or other mental illnesses were recruited. Groups were matched for age and premorbid intelligence using the Wechsler Test of Adult Reading. All participants were female, English-speaking, from the same geographical region, and had no history of significant brain injury or neurological disorder. Demographic and clinical information including age, body mass index (BMI), age of illness onset, duration of illness, and Eating Disorder Examination Questionnaire (EDE-Q) were obtained and recorded for each participant. EDE-Q is a self-reported assessment that examines the psychological and behavioral burden of AN.

### Image acquisition

All brain MRI scans were acquired using a 3-Tesla Siemens Tim Trio scanner with a 32-channel head coil at Swinburne University of Technology (Melbourne, Australia). No software or hardware updates were conducted during the course of data acquisition. T1 structural scans were acquired using an MPRAGE sequence with the following parameters: voxel size = 1 mm isotropic, TE = 2.52 ms, TR: 1900 ms, flip angle = 9°. For QSM estimation, Gradient Echo (GRE) images were acquired with TR = 44 ms, number of echoes = 7, TE1 = 10 ms, ΔTE = 5 ms, flip angle = 15°.

### Image processing

#### T1 structural images

To acquire a brain-mask, we used the Multi-Atlas Brain Segmentation (MABS) tool (https://github.com/pnlbwh/PNL-manual#multi-atlas-brain-segmentation-mabs) on Enterprise Research Infrastructure & Services (ERIS) computational network at Partners HealthCare. Based on the existing evidence on the volume decline in the thalamus, caudate nucleus, putamen, nucleus accumbens, hippocampus, and amygdala, we chose these subcortical structures as the regions of interest (ROI) for the ROI-based comparison of volume and QSM. We used Functional Magnetic Resonance Imaging of the Brain centre (FMRIB) Software Library (FSL) (FMRIB, Oxford University, UK) FIRST tool to segment the thalamus, caudate nucleus, putamen, and nucleus accumbens, and FreeSurfer v.7.1.0. for the segmentation of the hippocampus and amygdala. Visual quality control of the segmentations was performed by an investigator blinded to the group assignments.

#### QSM processing

QSM provides an accurate estimation of magnetic susceptibility at the voxel level. Magnetic susceptibility is a physical property that quantifies the magnetic field generated by matters in the presence of an external magnetic field (i.e. the extent to which matters become magnetized when exposed to a magnetic field). Substances such as water and myelin (the most abundant constituents of the brain tissue) are diamagnetic and have negative magnetic susceptibility. Iron and iron-containing molecules such as ferritin and neuromelanin are strongly paramagnetic and have positive magnetic susceptibility [9]. The intensity of QSM contrast in each voxel represents the algebraic summation of all positive and negative contributions to magnetic susceptibility, and is used as an indirect measure of iron content especially in the subcortical gray matter [12, 19]. Measured magnetic susceptibility can be positive or negative depending on the dominant contribution from iron or myelin and water.

In this study, QSM images were constructed using the Quantitative Susceptibility Mapping Artifact Reduction Technique (QSMART) pipeline [42] from the GRE acquisitions. In the first step, brain masks were created from the magnitude component of the GRE scan using the Brain Extraction Tool, FMRIB Software Library (FSL) (FMRIB, Oxford University, UK) [14]. Then, the phase component of the GRE scan was unwrapped using a Laplacian-based method [20]. The background field was then removed using a three-dimensional spatially dependent filtering [25], followed by a magnitude-weighted least squares method for the final dipole inversion step. A detailed description of this pipeline can be found in Yaghmaie et al. [42].

### Voxel-wise comparison of QSM between groups

To perform voxel-wise comparisons, QSM images were warped to a common template. In the first step, a study template was constructed from the skull-stripped T1 MRI images of all participants using the antsMultivariateTemplateConstruction2 tool, Advanced Normalization Tools software package (ANTs) v.2.3.5 (https://github.com/ANTsX/ANTs/) [3]. An affine and nonlinear transformation to the common template was created for each participant by warping their skull-stripped T1 image to the study template using the antsRegistrationSyN tool from ANTs. A rigid transformation from the GRE magnitude component to the T1 image for each participant was also created. Finally, the QSM image from each participant was warped to a common space by applying the above transformations sequentially. Voxel-wise comparisons were then conducted between each AN group and the control group using the randomise tool from FSL package controlling for age with 5000 permutations and variance smoothing using a 5 mm kernel. Threshold-Free Cluster Enhancement (TFCE) [36] method was used for Family-Wise Error Rate (FWER) adjustment [40].

### Statistical analysis

Age and BMI were compared between groups using independent samples *t* test. Volume and mean QSM value in the ROIs were compared between groups as the primary analyses using the analysis of covariance (ANCOVA) test with 5000 bootstrapping iterations. Estimated total intracranial volume (eTIV) was considered as a covariate in the comparison of ROI volume between groups. ANCOVA test of mean ROI QSM was conducted controlling for age. Adjustment for multiple comparisons was conducted for the primary analyses using the Benjamini–Hochberg method with a False Detection Rate of 0.05.

In each group, we further explored the correlation between QSM in each ROI and AN-associated variables including BMI, EDE-Q, illness duration, and age of illness onset. These correlations were assessed using the Pearson’s partial correlation test controlling for age. For these secondary analyses, confidence intervals were calculated with 5000 bootstrapping iterations. All statistical tests were conducted using IBM SPSS v. 24.

## Results

Neuroimaging and clinical data from the three groups of c-AN (n = 20), wr-AN (n = 16), and healthy controls (n = 18) were analyzed. All participants were female, and the study groups did not differ in age. Descriptive and comparative statistics (where applicable) of age, BMI, EDE-Q, AN duration and age of onset are presented in Table 1.

**Table 1:** Descriptive and comparative statistics of demographic and clinical characteristics of participants

	c-AN	wr-AN	Control	*p* value (c-AN vs. control)	*p* value (wr-AN vs. control)	*p *value (c-AN vs. wr-AN)
Age (SD) years	23.3 (4.4)	22.7 (4.1)	23.4 (3.8)	0.94	0.6	0.67
BMI (SD)	16.7 (1.5)	22.4 (3)	23.2 (3.2)	< 0.001	< 0.001	< 0.001
EDE-Q (SD)	3.9 (1.2)	2.6 (1.9)	0.7 (0.7)	< 0.001	< 0.001	0.03
Age of AN onset (SD) (years)	16 (3.1)	14.3 (2.6)	–	–	–	0.08
Duration of AN (SD) (years)	6.6 (5.4)	4.7 (4.1)	–	–	–	0.26

### Between-group comparison of ROI volumes

Consistent with the existing literature, reduced subcortical volume was observed in each AN group compared to the healthy controls, however, these differences in ROI volume did not survive correction for multiple comparisons. See Additional file 1 for details.

### Voxel-wise QSM comparison

Voxel-wise analysis to compare QSM in the whole brain did not reveal any differences between groups.

### ROI-based comparison of QSM between groups

**Fig. 1:**
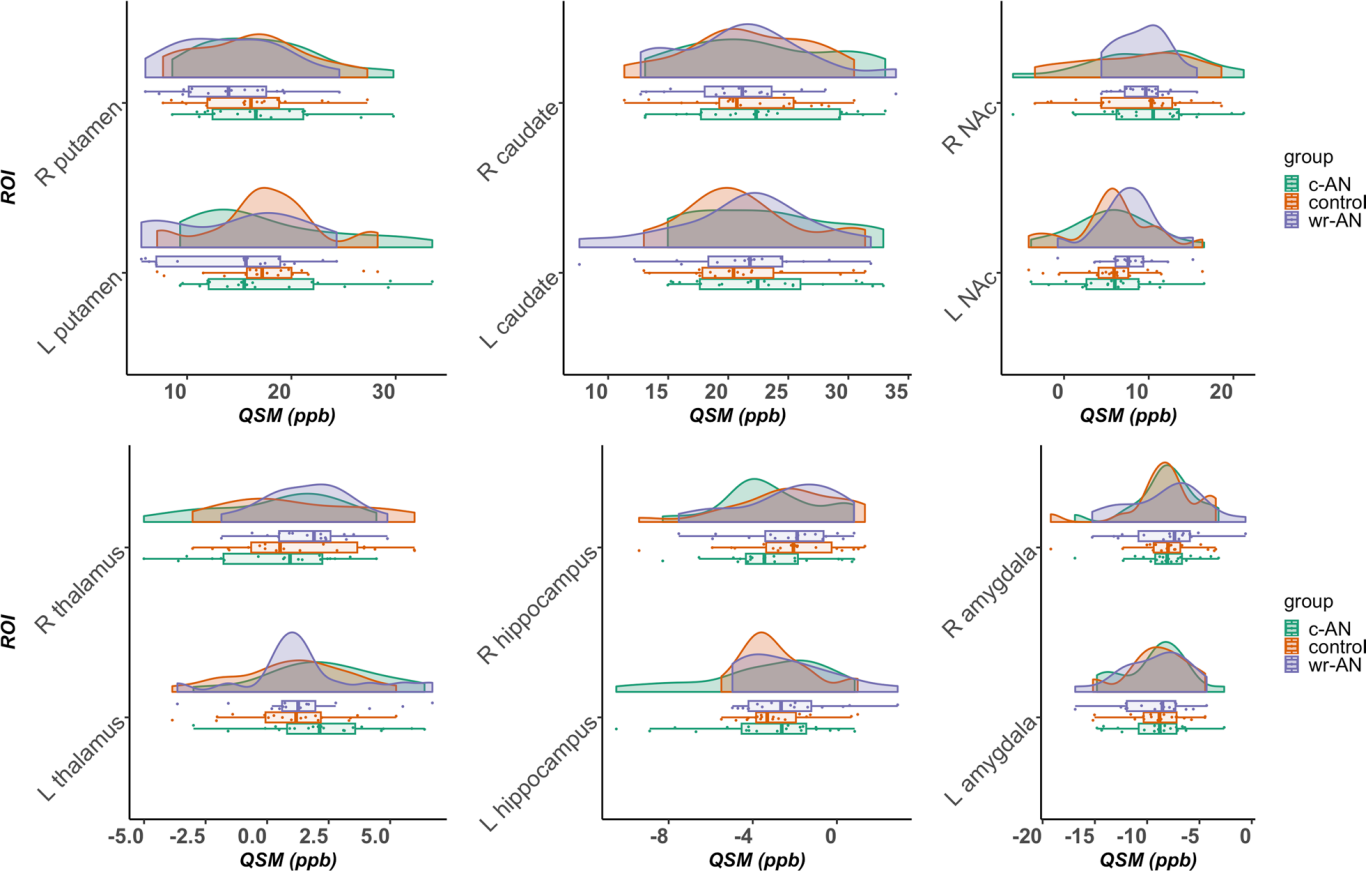
Raincloud plots of ROI QSM values across groups. In the box and whisker plots, boxes show the interquartile range and the line inside each box indicates the median value; the left and right whiskers show the lower and upper adjacent datapoints respectively

ROI-based comparison of QSM did not reveal any differences in the examined ROIs across groups. Detailed statistics and data visualization for the between-group comparison of QSM values are provided in Table 2 and Fig. 1.

**Table 2:** Between-group comparison of QSM controlled for age

QSM	Mean (SD) QSM, ppb			Three-way ANCOVA	c-AN versus control post-hoc		wr-AN versus control post-hoc		c-AN versus wr-AN post-hoc	
ROIs	c-AN	wr-AN	Control	*p*	*p*	Effect size (η_p_^2^)	*p*	Effect size (η_p_^2^)	*p*	Effect size (η_p_^2^)
Left putamen	17.6 (7.1)	14.3 (6.4)	17.5 (5.5)	0.24	0.89	0.00	0.19	0.06	0.16	0.06
Right putamen	17.1 (5.5)	14 (5.1)	16 (5.4)	0.24	0.43	0.02	0.36	0.03	0.11	0.08
Left caudate	22.8 (5.9)	21.1 (6)	21.3 (5.2)	0.62	0.36	0.02	0.92	0.00	0.46	0.02
Right caudate	22.6 (6.2)	21.1 (5.7)	21.7 (5.2)	0.77	0.60	0.01	0.88	0.00	0.51	0.01
Left NAc	5.7 (4.8)	7.6 (3.7)	5.7 (4.9)	0.36	0.97	0.00	0.20	0.06	0.19	0.05
Right NAc	9.8 (6.7)	9.3 (3.1)	8.1 (6.7)	0.69	0.45	0.02	0.55	0.01	0.74	0.00
Left thalamus	2.2 (2.4)	1.5 (2.5)	1 (2.3)	0.27	0.11	0.07	0.43	0.02	0.47	0.02
Right thalamus	0.4 (2.6)	1.6 (1.7)	1.2 (2.8)	0.22	0.33	0.03	0.49	0.01	0.05	0.11
Left hippocampus	− 3.5 (3.1)	− 2.4 (2.2)	− 2.9 (1.7)	0.43	0.42	0.02	0.56	0.01	0.23	0.04
Right hippocampus	− 3.1 (2.4)	− 2.4 (2.4)	− 2.4 (2.7)	0.60	0.35	0.03	0.86	0.00	0.47	0.02
Left amygdala	− 9.2 (3.1)	− 9.3 (3.2)	− 9 (3)	0.97	0.83	0.00	0.83	0.00	0.99	0.00
Right amygdala	− 8.2 (3.1)	− 8.2 (3.7)	− 8.2 (3.6)	1.00	0.99	0.00	0.96	0.00	0.94	0.00

η_p_^2^ = partial eta squared, NAc = nucleus accumbens

### Correlation between clinical indices and QSM

The results of correlation tests between clinical indices and QSM in the ROIs are detailed in Table 3. In these exploratory tests, correction for multiple comparisons has not been conducted and 95% confidence intervals (95% CI) are reported in lieu of *p* values to avoid misinterpretation. 95% CI ranges that do not contain the neutral value (zero) are considered statistically meaningful.

**Table 3:** Partial correlations between mean ROI QSM and clinical measures controlled for age with 5000 permutations

Clinical measure	ROIs	c-AN		wr-AN		Control	
		r	95% CI	r	95% CI	r	95% CI
BMI	Left putamen	0.01	(− 0.38, 0.44)	0.05	(− 0.57, 0.41)	0.24	(− 0.24, 0.66)
	Right putamen	− 0.14	(− 0.51, 0.31)	0.03	(− 0.61, 0.45)	0.33	(− 0.34, 0.69)
	Left caudate	− 0.07	(− 0.41, 0.28)	− 0.06	(− 0.47, 0.24)	0.32	(− 0.13, 0.68)
	Right caudate	− 0.21	(− 0.6, 0.22)	− 0.002	(− 0.38, 0.28)	0.32	(− 0.04, 0.69)
	Left NAc	0.12	(− 0.27, 0.49)	− 0.03	(− 0.51, 0.3)	− 0.15	(− 0.75, 0.45)
	Right NAc	0.08	(− 0.29, 0.44)	− 0.11	(− 0.58, 0.21)	− 0.08	(− 0.57, 0.45)
	Left thalamus	0.19	(− 0.21, 0.54)	− 0.34	(− 0.77, 0.57)	− 0.10	(− 0.62, 0.47)
	Right thalamus	0.02	(− 0.31, 0.39)	− 0.29	(− 0.68, 0.16)	0.14	(− 0.27, 0.57)
	Left hippocampus	− 0.36	(− 0.62, − 0.08)	− 0.39	(− 0.75, 0.14)	− 0.006	(− 0.58, 0.54)
	Right hippocampus	− 0.37	(− 0.74, 0.13)	− 0.5	(− 0.85, 0.03)	0.2	(− 0.27, 0.71)
	Left amygdala	− 0.32	(− 0.57, − 0.01)	− 0.4	(− 0.79, 0.74)	− 0.21	(− 0.55, 0.2)
	Right amygdala	− 0.29	(− 0.63, 0.06)	0.02	(− 0.42, 0.68)	0.06	(− 0.32, 0.52)
EDEQ	Left putamen	− 0.03	(− 0.51, 0.46)	− 0.3	(− 0.69, 0.17)		
	Right putamen	− 0.03	(− 0.52, 0.48)	− 0.2	(− 0.68, 0.24)		
	Left caudate	0.05	(− 0.44, 0.47)	− 0.43	(− 0.78, 0.17)		
	Right caudate	− 0.11	(− 0.53, 0.39)	− 0.29	(− 0.72, 0.2)		
	Left NAc	− 0.18	(− 0.63, 0.29)	− 0.35	(− 0.7, 0.25)		
	Right NAc	− 0.31	(− 0.72, 0.18)	0.13	(− 0.42, 0.6)		
	Left thalamus	− 0.09	(− 0.45, 0.22)	0.02	(− 0.55, 0.58)		
	Right thalamus	− 0.21	(− 0.53, 0.17)	0.33	(− 0.28, 0.8)		
	Left hippocampus	− 0.02	(− 0.41, 0.41)	− 0.46	(− 0.8, 0.05)		
	Right hippocampus	− 0.02	(− 0.43, 0.46)	− 0.33	(− 0.75, 0.14)		
	Left amygdala	0.58	(0.11, 0.82)	− 0.4	(− 0.78, 0.13)		
	Right amygdala	0.31	(− 0.25, 0.67)	− 0.23	(− 0.67, 0.34)		
AN age of onset	Left putamen	0.06	(− 0.45, 0.52)	− 0.33	(− 0.75, 0.2)		
	Right putamen	− 0.15	(− 0.62, 0.41)	− 0.23	(− 0.66, 0.29)		
	Left caudate	− 0.03	(− 0.59, 0.39)	− 0.44	(− 0.79, 0.31)		
	Right caudate	− 0.07	(− 0.53, 0.38)	− 0.35	(− 0.71, 0.27)		
	Left NAc	0.09	(− 0.44, 0.5)	− 0.3	(− 0.71, 0.53)		
	Right NAc	− 0.1	(− 0.63, 0.3)	− 0.04	(− 0.68, 0.62)		
	Left thalamus	− 0.41	(− 0.77, − 0.14)	0.22	(− 0.43, 0.68)		
	Right thalamus	− 0.1	(− 0.59, 0.28)	0.27	(− 0.36, 0.77)		
	Left hippocampus	− 0.39	(− 0.82, − 0.13)	− 0.15	(− 0.62, 0.38)		
	Right hippocampus	− 0.5	(− 0.89, − 0.19)	− 0.28	(− 0.68, 0.39)		
	Left amygdala	0.1	(− 0.38, 0.52)	− 0.06	(− 0.65, 0.46)		
	Right amygdala	0.002	(− 0.46, 0.3)	− 0.27	(− 0.67, 0.4)		
Duration of AN	Left putamen	− 0.13	(− 0.54, 0.32)	0.52	(− 0.14, 0.77)		
	Right putamen	0.04	(− 0.48, 0.53)	0.51	(− 0.17, 0.74)		
	Left caudate	− 0.01	(− 0.43, 0.47)	0.45	(− 0.1, 0.8)		
	Right caudate	0.09	(− 0.34, 0.64)	0.39	(− 0.28, 0.89)		
	Left NAc	− 0.17	(− 0.58, 0.31)	0.36	(− 0.54, 0.71)		
	Right NAc	0.22	(− 0.2, 0.66)	0.03	(− 0.46, 0.74)		
	Left thalamus	0.46	(0.2, 0.75)	− 0.08	(− 0.57, 0.46)		
	Right thalamus	0.06	(− 0.32, 0.51)	0.03	(− 0.61, 0.51)		
	Left hippocampus	0.31	(0, 0.73)	0.06	(− 0.5, 0.56)		
	Right hippocampus	0.24	(− 0.33, 0.8)	0.27	(− 0.21, 0.74)		
	Left amygdala	− 0.22	(− 0.57, 0.25)	− 0.05	(− 0.47, 0.48)		
	Right amygdala	− 0.02	(− 0.37, 0.51)	0.01	(− 0.63, 0.56)		

No adjustment for multiple comparison was performed for these exploratory tests

Among the three participant groups, we observed meaningful correlations only in the c-AN group. Lower BMI correlated with higher QSM in the left hippocampus [r = − 36, 95% CI (− 0.62, − 0.08)] and left amygdala [r = − 0.32, 95% CI (− 0.57, − 0.01)]; higher EDE-Q scores, indicative of higher illness severity, correlated with higher QSM in the left amygdala [r = 0.58, 95% CI (0.11, 0.82)]; longer duration of illness correlated with higher QSM in the left thalamus [r = 0.46, 95% CI (0.2, 0.75)] and left hippocampus [r = 031, 95% CI 0, 0.73)]; younger age of onset correlated with higher QSM in the left thalamus [r = − 0.41, 95% CI (− 0.77, − 0.14)] and bilateral hippocampus [left: r = − 0.39, 95% CI (− 0.82, − 0.13), right: r = − 0.5, 95% CI (− 0.89, − 0.19)]. In the wr-AN and healthy control groups, 95% CI for all correlation tests contained both positive and negative values.

## Discussion

In the first study of its kind, we used QSM to investigate brain iron alterations in individuals with AN. The ROIs selected as the primary focus of this study were the putamen, caudate nucleus, nucleus accumbens, thalamus, hippocampus and amygdala. Compared to healthy controls, ROI volumes were lower in the right putamen and right thalamus in c-AN, and in the right caudate nucleus, left nucleus accumbens and left thalamus in wr-AN. Although these between-group comparisons did not survive statistical correction for multiple comparisons, our observed effect sizes for the comparison between AN and control groups are comparable [18] with a recent, large-scale ENIGMA consortium study [38]. In the QSM comparisons, neither the whole-brain voxel-wise analysis, nor the ROI-based comparison revealed any differences between AN and control groups. This finding does not support our hypothesis and indicates no alteration in brain iron content in either the acute emaciation phase or the weight-restored stage of AN. Importantly, the effect sizes for the difference in QSM between AN groups and healthy controls were predominantly small (partial Eta squared ≤ 0.03), and smaller than the effect sizes observed for the comparison of ROI volumes in this same population. Therefore, our data suggests that a direct impact of AN on brain iron is unlikely. The results of our exploratory correlational analyses, however, may implicate a secondary or mediatory link between iron and the neurobiology of AN.

Our exploratory analyses examined the association between regional brain iron content and clinical indices in each AN group. These correlations were statistically meaningful only in the c-AN group. More severe phenotypes, in both EDE-Q and BMI, were associated with higher iron in the left hippocampus and left amygdala. Furthermore, longer duration of AN and younger age of onset were both associated with higher iron content in the left thalamus and left hippocampus. These findings collectively suggest a link between longer course and higher severity of AN with an increased regional brain iron content and point to a potential longitudinal accumulation of iron over the course of AN. These results are inconsistent with the expectation that the progression of AN would be associated with poorer nutrition and hence produce iron deficiency.

The effect of AN on the brain is multifactorial and mediated by an interaction of cell dehydration, micro-/macronutrient deficiencies, neural cell loss, and biological processes relevant to hormonal changes (discussed in detail in [17]). These variables can also affect brain iron [10]. As reported in neurodegenerative diseases, excess iron generates oxidative stress and leads to neurotoxicity [23]. Thus, a possible longitudinal and progressive accumulation of iron may play a role in the volume loss observed in AN brains. Since a neurotoxic effect from iron would be mediated by oxidative stress, the concurrent use of QSM and magnetic resonance spectroscopy (MRS), which allows for examining oxidative stress, can provide valuable insights into whether brain iron accumulation in AN would be associated with greater oxidative stress and therefore neurotoxicity.

Our findings cannot inform on the causality of the relationship between AN and brain iron. However, the presence of such correlations only in the c-AN (and not the wr-AN) group suggests that the association between brain iron and these clinical indices may be partially mediated by the effect of body weight and calorie intake. Further, considering the evidence of increased levels of the iron regulatory proteins, ferritin and hepcidin responsible for promoting the storage of iron in the tissues [26], these observed correlations could be explained by an increased ferritin-bound iron deposition in the brain. Longitudinal studies examining brain iron during current and weight-restored phases of AN would provide a clearer picture of such associations.

The present study has certain limitations. One, is the relatively small sample size which limits the statistical power for detection of small effect sizes. Another shortcoming was the lack of concurrent serum iron measurement to assess for the systemic iron status in our study population. The availability of serum iron indices would allow for the evaluation of brain iron while accounting for (and examining its potential link with) systemic indices such as serum iron, ferritin, and hepcidin levels. The third limitation was that in our study, the c-AN and wr-AN groups were separate cohorts, rather than a repeat assessment of the c-AN after recovery. A longitudinal design with multiple brain imaging through the course of AN from acute weight loss and nutrient/energy deficiency to weight restoration and re-establishment of the energy balance would allow for more accurate investigation of the association between AN and brain iron and minimize the confounding effect of individual variations. Finally, nutrition diary was not obtained in our study. The availability of such information would allow for the evaluation of the link between dietary iron and brain iron content.

## Conclusion

The present study provides a preliminary investigation of brain iron in AN. While no difference was observed in brain iron among groups, regional iron content correlated with a number of clinical indices in AN, especially BMI. Our findings warrant further studies that would provide a more comprehensive understanding of brain iron dysregulation in AN and how pathophysiological brain differences are involved in the disorder.

### Supplementary Information

**Additional file 1**. Detailed statistal and graphic report on the volumetric comparison of ROIs across groups.

## Data Availability

The datasets that support the findings of this study consist of individuals’ neuroimaging data, which are not publicly available per the conditions of our Human Research Ethics Committee approval. Anonymized neuroimaging data and extracted metrics generated from processed data, however, will be made available upon request and a data sharing agreement.

## Abbreviations

AN: Anorexia nervosa
ANTs: Advanced Normalization Tools software package
BMI: Body mass index
c-AN: Current anorexia nervosa
EDE-Q: Eating Disorder Examination Questionnaire
ERIS: Enterprise Research Infrastructure and Services
eTIV: Estimated total intracranial volume
FMRIB: Functional magnetic resonance imaging of the brain centre
FWER: Family-Wise Error Rate
GRE: Gradient recalled echo
MABS: Multi-Atlas Brain Segmentation
MINI: Mini International Neuropsychiatric Interview
MRI: Magnetic resonance imaging
MRS: Magnetic resonance spectroscopy
QSM: Quantitative susceptibility mapping
QSMART: Quantitative susceptibility mapping artifact reduction technique
ROI: Region of interest
TFCE: Threshold-Free Cluster Enhancement
wr-AN: Weight-restored anorexia nervosa
