# Supplementary material for: Investigation of brain iron in anorexia nervosa, a quantitative susceptibility mapping study
Source: J Eat Disord. 2023 Aug 21;11:142. doi: 10.1186/s40337-023-00870-4 (PMC10441741; doi:10.1186/s40337-023-00870-4)
Supplement: Supplementary file 1 — Additional file 1. Detailed statistal and graphic report on the volumetric comparison of ROIs across groups. [file 40337_2023_870_MOESM1_ESM.docx]

## Supplementary material 1.

### Between-group comparison of ROI volumes

In the c-AN group, right putamen and right thalamus had lower volumes compared to healthy controls. The right caudate nucleus, left nucleus accumbens and left thalamus were smaller in the wr-AN group than healthy controls. However, these differences did not survive correction for multiple comparisons. Further, there was no difference in the volume of any of the ROIs between c-AN and wr-AN groups. Descriptive and comparative statistics for ROI volumes are presented in Table S1 and plotted in Figure S1.

Table S1. Between group comparison of ROI volume controlled for eTIV. ANCOVA with 5000 bootstrapping iterations. p-values provided in this table are raw values. None of the significant unadjusted p-values survived correction for multiple comparisons.

| volume | mean volume (SD), mm^3^ | | | three-way ANCOVA | c-AN vs. control post-hoc | | wr-AN vs. control post-hoc | | c-AN vs. wr-AN post-hoc | | |  |
| --- | --- | --- | --- | --- | --- | --- | --- | --- | --- | --- | --- | --- |
| ROIs | c-AN | wr-AN | control | p | p | effect size (η_p_^2^) | p | effect size (η_p_^2^) | p | effect size (η_p_^2^) |  |  |
| left putamen | 4847 (370.8) | 5114.5 (464.8) | 5097.4 (565) | 0.20 | 0.11 | 0.07 | 0.89 | 0.00 | 0.13 | 0.07 |  |  |
| right putamen | 4963.1 (351.1) | 5133.7 (384.9) | 5300.3 (587.7) | 0.07 | 0.04 | 0.12 | 0.19 | 0.05 | 0.34 | 0.03 |  |  |
| left caudate | 3676.9 (336.5) | 3634.3 (383.4) | 3783.1 (401.6) | 0.26 | 0.41 | 0.02 | 0.10 | 0.09 | 0.38 | 0.02 |  |  |
| right caudate | 3746.7 (311.9) | 3582.1 (447.8) | 3839.9 (435.4) | 0.05 | 0.51 | 0.01 | 0.03 | 0.16 | 0.08 | 0.09 |  |  |
| left NAc | 528.4 (80.2) | 519.8 (90.2) | 587.1 (105.3) | 0.04 | 0.05 | 0.10 | 0.03 | 0.15 | 0.52 | 0.01 |  |  |
| right NAc | 461.1 (102.8) | 438.7 (67.3) | 473.1 (84.5) | 0.35 | 0.74 | 0.00 | 0.16 | 0.07 | 0.27 | 0.03 |  |  |
| left thalamus | 7966.9 (666.2) | 8010.2 (719.1) | 8325.9 (667.3) | 0.06 | 0.06 | 0.09 | 0.02 | 0.15 | 0.59 | 0.01 |  |  |
| right thalamus | 7758.8 (649.1) | 7924.5 (699.6) | 8129.4 (607) | 0.08 | 0.03 | 0.12 | 0.07 | 0.10 | 0.87 | 0.00 |  |  |
| left hippocampus | 4215.9 (351.1) | 4155.8 (298.3) | 4285 (310.6) | 0.24 | 0.58 | 0.01 | 0.08 | 0.08 | 0.24 | 0.05 |  |  |
| right hippocampus | 4533.1 (379.2) | 4477.4 (352) | 4581.7 (415.2) | 0.30 | 0.81 | 0.00 | 0.11 | 0.08 | 0.19 | 0.05 |  |  |
| left amygdala | 1567.7 (180.2) | 1621.1 (196.1) | 1635.1 (214.5) | 0.58 | 0.29 | 0.03 | 0.58 | 0.01 | 0.69 | 0.01 |  |  |
| right amygdala | 1707.8 (169.1) | 1748.1 (205) | 1813.7 (227) | 0.17 | 0.07 | 0.09 | 0.15 | 0.07 | 0.91 | 0.00 |  |  |
| η_p_^2^ = partial Eta squared | | | | | | | | | | | | |
| eTIV = estimated total intracranial volume | | | | | | | | | | | | |
| NAc = nucleus accumbens | | | | | | | | | | | | |


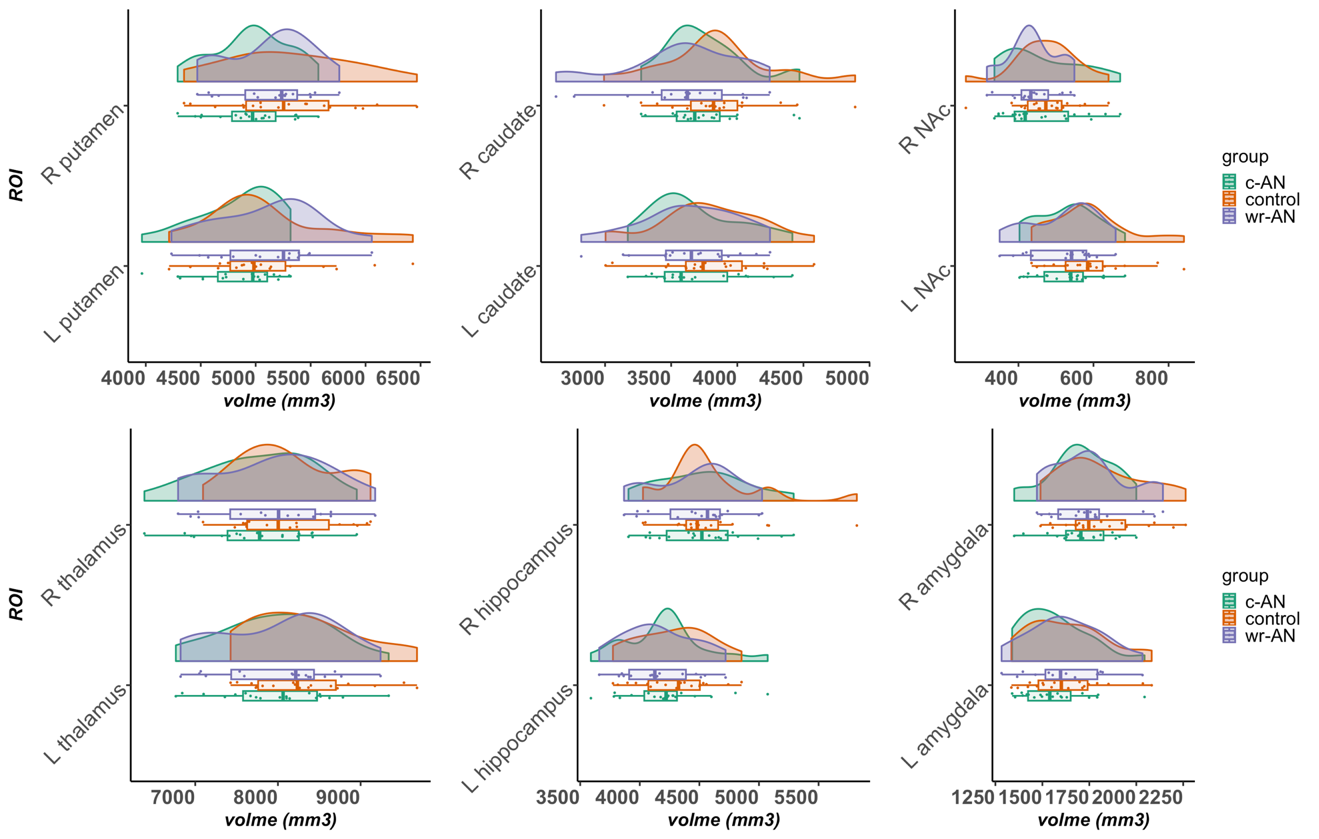


Figure S1. raincloud plots of ROI volumes across groups. In the box and whisker plots, boxes show the interquartile range and the line inside each box indicates the median value; the left and right whiskers show the lower and upper adjacent datapoints respectively. Data points outside whiskers are considered statistical outliers.
